# The Impact of Coronavirus Disease 2019 Epidemic on Dizziness/Vertigo Outpatients in a Neurological Clinic in China

**DOI:** 10.3389/fneur.2021.663173

**Published:** 2021-04-29

**Authors:** Changqing Li, Dongsheng Guo, Xiangke Ma, Siwei Liu, Mingyong Liu, Lichun Zhou

**Affiliations:** ^1^Department of Neurology, Beijing Chaoyang Hospital, Capital Medical University, Beijing, China; ^2^Department of Emergency, Beijing Chaoyang Hospital, Capital Medical University, Beijing, China; ^3^Department of Neurosurgery, Beijing Chaoyang Hospital, Capital Medical University, Beijing, China

**Keywords:** COVID-19, dizziness/vertigo, BPPV, psychogenic/PPPD, China

## Abstract

**Objective:** This study aims to investigate the impact of the coronavirus disease 2019 (COVID-19) epidemic on dizziness/vertigo outpatients in a neurological clinic in China.

**Methods:** Against the background of the COVID-19 epidemic, the data of patients who visited the neurological clinic of Beijing Chaoyang Hospital West Branch during the pandemic (February 1–May 30, 2020) and the corresponding period in 2019 (February 1–May 30, 2019) were analyzed, and patients with dizziness/vertigo from these two periods were compared to discover their demographic features and etiologic distribution according to their age and sex.

**Result:** The absolute number of neurological outpatients decreased from 14,670 in 2019 to 8,763 in 2020 (−40.3%), with a corresponding decline in dizziness/vertigo patients (2019: *n* = 856; 2020: *n* = 1,436, −40.4%). Dizziness/vertigo was more common in women than men in these two periods (2019: women = 63.6%; 2020: women = 63.1%, *p* = 0.82). The overall etiology distribution was different among all disorders between the two periods (*p* < 0.001). There was an increase in benign paroxysmal positional vertigo (BPPV) (2019 vs. 2020: 30.7 vs. 35%, *p* < 0.05) and psychogenic/persistent postural perceptual dizziness (PPPD) (2019 vs. 2020: 28.5 vs. 34.6%, *p* < 0.05) while a decrease in vascular vertigo during the epidemic (2019 vs. 2020: 13 vs. 9.6%, *p* < 0.05). During the epidemic, the top three causes of dizziness/vertigo were BPPV (35%), psychogenic/PPPD (34.6%), and vascular vertigo (9.6%). A female predominance was observed in BPPV (women = 67.7%, *p* < 0.05) and psychogenic/PPPD (women = 67.6%, *p* < 0.05). In addition, the etiology ratio of different age groups was significantly different (*p* < 0.001). The most common cause for young and young-old patients was BPPV, and the most common cause for middle-aged and old-old patients was psychogenic/PPPD.

**Conclusion:** The absolute number of outpatients with dizziness/vertigo during the COVID-19 pandemic was reduced during the early period of the COVID-19 outbreak. BPPV and psychogenic/PPPD were more abundant, and vascular vertigo was less frequent. Based on those data, health-care management policy for dizziness/vertigo and mental disorder should be developed during the outbreak of COVID-19 and other infective diseases.

## Introduction

As a sudden acute respiratory infectious disease, coronavirus disease 2019 (COVID-19) imposed such a great impact on public health that people's focus in life underwent tremendous changes overnight. During the early phase of the COVID-19 outbreak, people spent less time outdoors, and tried to stay away from public places such as hospitals. Moreover, the urgent diversion of medical staff and hospital resources to COVID-19 emergencies inevitably severely compromised normal medical care. Dizziness/vertigo as a subjective and non-specific symptom is frequently complained by outpatients in the neurology department, with high incidence and recurrence rates. Does the COVID-19 epidemic affect the occurrence of dizziness/vertigo? This study compared the demographic characteristics and etiological distribution of dizziness/vertigo outpatients in a neurological clinic during the COVID-19 epidemic in 2020 and the same period in 2019 in order to assess the possible relationship between these changes and occurrence of COVID-19.

## Methods

### Subjects

The data of all outpatients and dizziness/vertigo outpatients in the neurological clinic of the West Branch of Beijing Chaoyang Hospital were retrospectively and continuously collected, which included 856 dizziness/vertigo patients aged 19–90 during the epidemic period (from February 1 to May 30, 2020) and 1,436 dizziness/vertigo patients aged 18–92 during the same period in 2019 (from February 1 to May 30, 2019).

Information covers the characteristics of dizziness/vertigo attacks (including duration, provoking factors, frequency, accompanying symptoms, and comorbidities) and detailed medical records including the patient's vital signs, nervous system examination, neuro-otological examination, vestibular function tests [for example, electronystagmography, caloric vestibular test, pure-tone audiometry, and head impulse–nystagmus–test of skew (HINTS)] Hamilton anxiety and depression scale, magnetic resonance imaging of the head or internal auditory canal, magnetic resonance angiography or CT of the head, cervical spine X-ray or MRI, carotid ultrasound, subclavian artery ultrasound, cardiac ultrasound, routine blood tests, and blood biochemistry. Based on these, the patients' demographic characteristics and etiological distribution according to their age and sex were analyzed and summarized.

### Diagnostic Criteria

The etiology of dizziness/vertigo was diagnosed according to the classification of International Classification of Vestibular Disorders (ICVD), including benign paroxysmal positional vertigo (BPPV) (1), psychiatric or persistent postural perceptual dizziness (psychogenic/PPPD) (2), vascular vertigo [caused by transient ischemic attack (TIA), acute cerebral infarction, cerebral hemorrhage, and cerebral small vascular disease] (3), vestibular migraine (VM) (4), vestibular neuritis (VN) (5), Mènière's disease (MD) (6), sudden sensorineural hearing loss (SSHL) accompanied by vertigo (7), systemic diseases (including hypertension, diabetes mellitus, postural hypotension, anemia, and cardiogenic diseases), and vestibular paroxysmia (VP) (8). Other causes include hereditary, metabolic, toxic, trauma-related, inflammatory, and demyelinating diseases. In addition, psychogenic dizziness and PPPD are not calculated separately because these two disorders are related to each other and often overlap (2).

### Statistical Analysis

The *t*-test was used to analyze quantitative variables such as the age difference between the two periods, and the chi-squared test was used to compare dizziness/vertigo patients during the COVID-19 period and the same period in 2019 in terms of etiologic distribution according to their age and sex. All statistical analyses were conducted using R statistical language (version 3.6.3, https://www.r-project.org/), and *p* < 0.05 was considered significant.

## Results

### Overall Comparison During the Two Periods

During the epidemic period, the absolute number of outpatients massively dropped (*n* = 8,763) compared with the same period in 2019 (*n* = 14,670, −40.3%), paralleling the decline in dizziness/vertigo patients (2019: *n* = 856; 2020: *n* = 1,436, −40.4%), while the relative dizziness/vertigo rate remained unchanged (9.7 vs. 9.7%, *p* = 0.97).

### Comparison of Demographic Features and Etiologic Distribution According to Their Age and Sex During Two Periods

Our data showed a female predominance (2019: women = 63.6%; 2020: women = 63.1%, *p* = 0.82) in both periods. The average age of dizziness/vertigo patients was 56.78 ± 14.12 years during the epidemic period and 55.97 ± 15.05 years during the same period in 2019. There is no statistically significant difference in age (*p* = 0.20).

During the epidemic period, BPPV was the most common cause, accounting for 35%, followed by psychogenic/PPPD (34.6%), and vascular vertigo (9.6%). These three diseases comprised 79.2% of all disorders. Notably, BPPV and psychogenic/PPPD accounted for nearly half of all disorders (see Table 1).

**Table 1:** The etiologic distribution of 856 dizziness/vertigo patients during the COVID-19 epidemic.

**Diagnosis**	***n* (%)**
BPPV	300 (35)
psychogenic/PPPD	296 (34.6)
Vascular vertigo	82 (9.6)
VM	33 (3.9)
VN	6 (0.7)
MD	8 (0.9)
SSHL accompanied by vertigo	9 (1.1)
Systemic disease	74 (8.6)
VP	5 (0.6)
Other causes	43 (5)

*COVID-19, coronavirus disease 2019; BPPV, benign paroxysmal positional vertigo; VM, vestibular migraine; VN, vestibular neuritis; MD, Mènière's disease; SSHL, sudden sensorineural hearing loss; VP, vestibular paroxysmia*.

**Figure 1:**
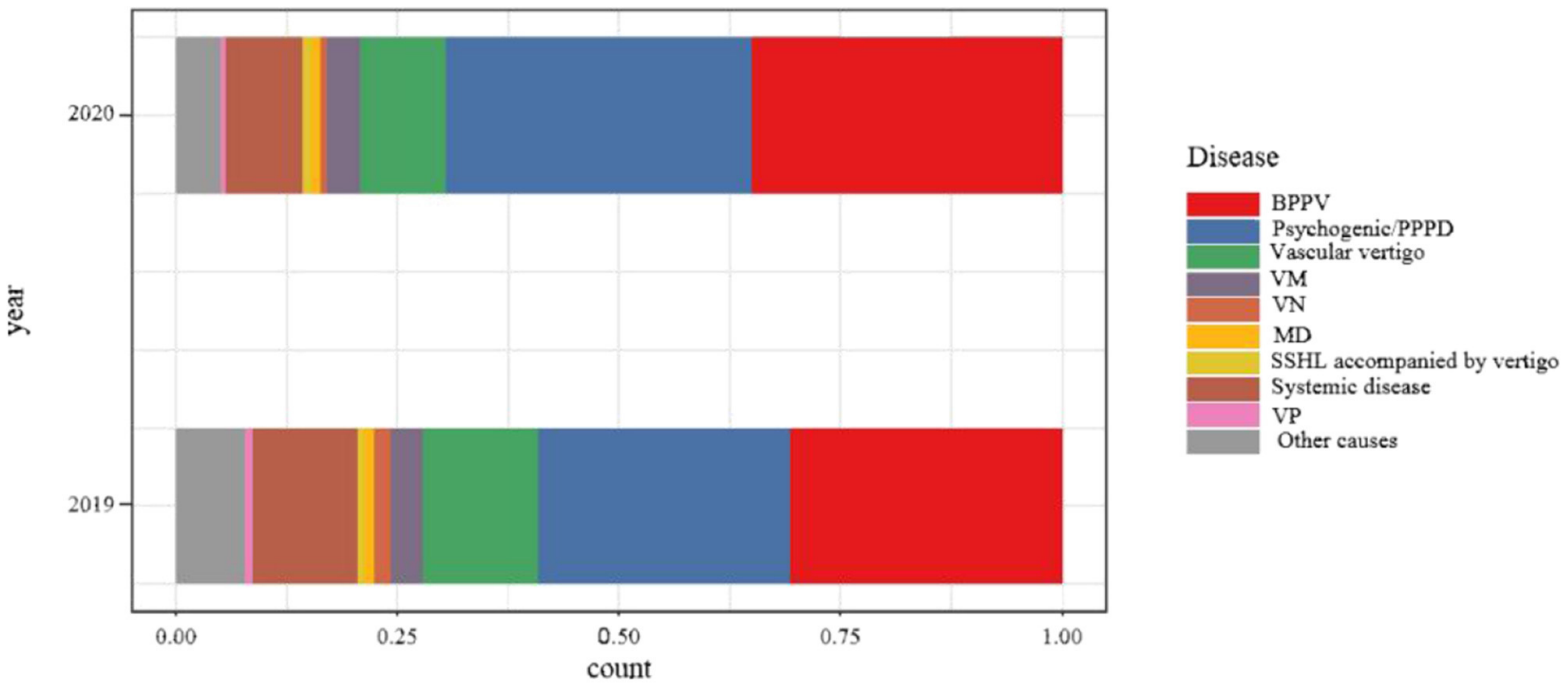
Comparison of etiologic distribution of patients with dizziness/vertigo of the study period in 2019 and 2020.

The overall etiology distribution was different among all disorders between the two periods (*p* < 0.001). There was an increase in BPPV (2019 vs. 2020: 30.7 vs. 35%, *p* < 0.05) and psychogenic/PPPD (2019 vs. 2020: 28.5 vs. 34.6%, *p* < 0.05) while a decrease in vascular vertigo during the epidemic (2019 vs. 2020: 13 vs. 9.6%, *p* < 0.05). In addition, the distribution of VM, VN, MD, sudden deafness with dizziness, dizziness caused by systemic diseases, VP, and other causes did not show a significant difference in the two-period comparison (*p* > 0.05) (see Figure 1).

### Sex Stratification of the Causes of Dizziness/Vertigo During Coronavirus Disease 2019

**Figure 2:**
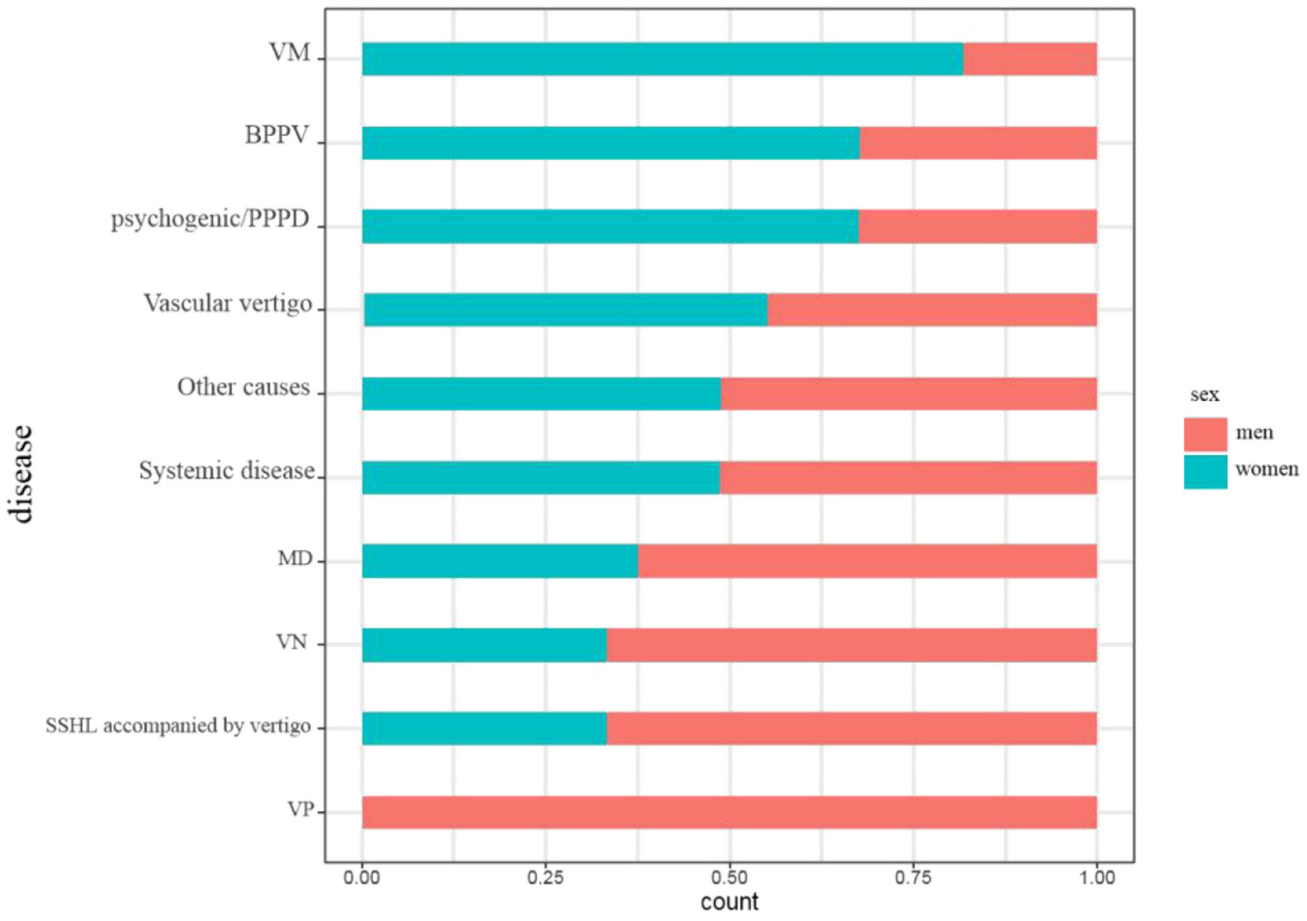
The sex ratios of each cause during the coronavirus disease 2019 (COVID-19) epidemic.

The sex ratio was significantly different among all disorders (*p* < 0.001). A female predominance was observed in BPPV (women = 67.7%, *p* < 0.05) and psychogenic/PPPD (women = 67.6%, *p* < 0.05). In contrast, the sex ratio of other causes did not show a significant difference (*p* > 0.05) (see Figure 2).

### Age Stratification of the Causes of Dizziness/Vertigo During Coronavirus Disease 2019

**Figure 3:**
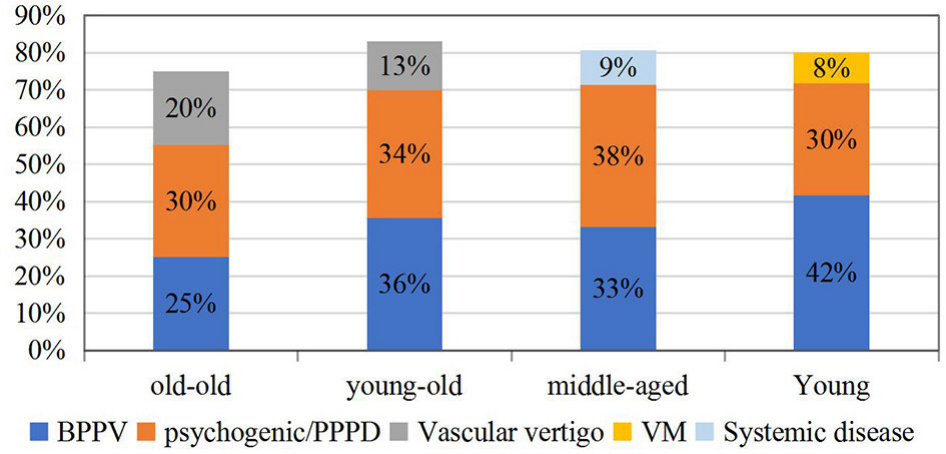
The etiologic distribution of dizziness/vertigo by the age group during the coronavirus disease 2019 (COVID-19) epidemic.

Our data showed that among all the causes, the patients with vascular vertigo were the oldest (56.52 ± 14.21), while those with VM were the youngest (48.79 ± 12.70). All patients were divided into four groups according to their age, namely, young (18–44 years old), middle-aged (45–59 years old), young-old (60–75 years old), and old-old (over 75 years old) groups. The etiology of 856 patients was stratified by age, and it was found that the etiology ratio of different age groups was significantly different (*p* < 0.001). The most common cause of young was BPPV, the most common cause of middle-aged was psychogenic/PPPD, the most common cause of young-old was BPPV, and the most common cause of old-old was psychogenic/PPPD (see Figure 3).

## Discussion

To our knowledge, this is the first study to examine the change in dizziness/vertigo outpatients in a neurological clinic. Our data showed a profound decrease in the number of neurology clinic visitors during the peak of the COVID-19 pandemic, paralleling a corresponding decline in the absolute number of dizziness/vertigo patients. Similar changes can also be observed in other neurological diseases. Many reports indicated that the COVID-19 outbreak impacted stroke care significantly all over the world, including a significant drop in acute stroke care, TIA, intravenous thrombolysis, and intracerebral hemorrhages (9–11). Fear of COVID-19 infection when visiting the hospital reduced the frequency of patients seeking medical help during the pandemic, and most patients opted for remote medical platforms for consultation. Besides, after the lockdown of Wuhan, strict isolation measures were implemented in Beijing, and the availability of traffic was also severely restricted. Meanwhile, social isolation may also have reduced the chance of identifying dizziness/vertigo among relatives and friends.

BPPV and psychogenic/PPPD are common causes of dizziness, and the reason for their relative increase in the early period of the COVID-19 outbreak lies in people's psychological stress response to the epidemic, resulting in emotional abnormalities such as anxiety/depression, insomnia, and acute stress. Shi et al. found that in the initial stage of the epidemic, among the Chinese population, depression accounted for 27.9%, anxiety 31.6%, insomnia 29.2%, and acute stress 24.4% (12). Xiang et al. also showed that major public health events or disasters, such as severe acute respiratory syndrome, bird flu, and Wenchuan earthquake, lead to a significant increase in the incidence of emotional problems during and after the occurrence (13). Meanwhile, a previous research revealed that anxiety and depression are common among patients with BPPV, and mood disorders increase the risk of the incidence of BPPV regardless of any gender and age (14, 15). Furthermore, from both anatomical and functional points of view, widespread vestibular projections to networks are involved in emotional processing (16).

The relative decrease of vascular vertigo during the pandemic corresponded to the similar change in stroke vascular events. However, these data only include statistics of hospitalized and emergency patients (9–11). Our data referred to face-to-face outpatients with mild symptoms and patients with severe or acute illness who often visit the emergency department, resulting in fewer patients with vascular vertigo. In addition, vascular vertigo in this study was diagnosed with not only TIA and stroke but also small cerebral vascular disease. A growing number of studies have found that unexplained dizziness, especially in the elderly, is often attributed to small cerebral vascular disease (17, 18).

In the case of vascular vertigo outpatients with confirmed or suspected stroke or TIA, epidemiological and fast-track COVID-19 screening was implemented. If intravenous thrombolysis is needed, the stroke team went into action to quickly initiate an optimal treatment plan and in-hospital consultation. Patients with confirmed or suspected COVID-19 infection should be isolated in the negative pressure carrier isolators and should undergo a special examination to determine risk–benefit ratio if intravenous thrombolysis and mechanical thrombectomy are initiated. Patients with non-COVID-19 infection must then be admitted to an isolated ward in the emergency department for intravenous thrombolysis. When mechanical thrombectomy is required, if the patient is diagnosed as confirmed or suspected COVID-19 infection by fast-track tests and the multidisciplinary team consultation, weighing the potential risks and benefits of treatment is essential, and patients with non-COVID-19 infection should follow routine surgical procedures and be admitted to the buffer ward after the operation, which was set up by the administrator of our hospital.

The low number of VN was remarkable in this study. At present, VN accounts for 0.5–9.0% in the vertigo clinic of the neurology department in China (19). Due to the lack of epidemiological data, insufficient understanding, and possible research selection bias, the incidence of this disorder may be underestimated. In addition, VN is characterized by rapid onset and long duration, so patients with VN are more likely to go to the emergency department for treatment, resulting in low incidence in the outpatient clinics.

Our data described a female preponderance among patients with dizziness/vertigo especially in BPPV and psychogenic/PPPD, which is in line with previous studies (20–22). The reason for the sex difference may be related to hormonal changes, differences in structure in the peripheral vestibular system, gender-based comorbidity, and so on (23, 24). Unfortunately, no sex difference was observed in vascular vertigo.

In addition to sex, age is also an important factor in the etiology of dizziness/vertigo. The causes of dizziness/vertigo at different ages had their characteristics. This study revealed that BPPV and psychogenic/PPPD were most prevalent in all disorders across all age groups during the COVID-19 epidemic, which is inconsistent with previous studies, especially in the old-old group (20, 25). The reason for this change might be the special stress caused by the epidemic. For the old-old, the social isolation during the epidemic posed a “serious public health problem” because it put them at greater risks of cardiovascular disease, autoimmune, neurocognitive, and mental health problems. Armitage et al. found that social isolation confronts the old-old with greater risks of depression and anxiety (26), so the incidence of BPPV and psychogenic/PPPD in the old-old group was also high.

It is well-known that the prevalence of BPPV increases with age (15, 20). Our data discovered that in the young (18–44 years old), BPPV (42%) was the most common, followed by PPPD. Recently, several studies have focused on the association of BPPV with the levels of 25(OH)D (27, 28), so its prevalence could be explained by lower 25(OH)D levels of both males and females aged <40 years in BPPV. Moreover, anxiety during the epidemic in the young increased the risk of BPPV (13).

This research has certain shortcomings. First of all, the data were a summary of neurological outpatients, so their characteristics may be different from those of general practice, otolaryngology, emergency, and other outpatients. Second, due to the lack of a sound general practitioner system in my country, a large number of patients with mental disorders related to dizziness visit the neurology department, which may be different from the results analyzed by foreign neurology and otolaryngology clinics. And lastly, although our center is a tertiary hospital in western Beijing, this study was a single-center study, which may be less representative.

## Conclusion

In short, we found a profound decline in the absolute numbers of outpatients with dizziness/vertigo in the neurological clinic during the COVID-19 pandemic. The etiological distribution of dizziness/vertigo during this period was different from that of the corresponding period in 2019. BPPV and psychogenic/PPPD were more abundant, and vascular vertigo was less frequent. Given the high incidence of BPPV and psychogenic/PPPD in the neurological clinic, clinicians should pay attention to the identification of these two causes, as well as the emotional disorders as a triggering factor that are likely to be caused by the epidemic. Maybe it is necessary to develop measures to improve health management of dizziness/vertigo and enhance mental health management during the outbreak of COVID-19 and other infective diseases.

## Data Availability Statement

The raw data supporting the conclusions of this article will be made available by the authors, without undue reservation.

## Ethics Statement

The studies involving human participants were reviewed and approved by Beijing Chaoyang Hospital. The patients/participants provided their written informed consent to participate in this study.

## Author Contributions

LZ, ML, and CL conceived and designed the study. CL, DG, and SL performed the study. XM and CL analyzed the data. CL wrote the paper. All authors reviewed and revised the manuscript critically for important intellectual content. All authors read and approved the final manuscript.

## Conflict of Interest

The authors declare that the research was conducted in the absence of any commercial or financial relationships that could be construed as a potential conflict of interest.
